# Modeling and Optimization of Infrared‐Convection Drying Parameters and Slice Thickness of Onion Slices: Effects on Drying Kinetics and Physicochemical Quality

**DOI:** 10.1002/fsn3.70437

**Published:** 2025-06-16

**Authors:** Hany S. El‐Mesery, Ahmed H. ElMesiry, Zicheng Hu, Xinai Zhang, Evans K. Quaye

**Affiliations:** ^1^ School of Energy and Power Engineering Jiangsu University Zhenjiang China; ^2^ Agricultural Engineering Research Institute Agricultural Research Center Dokki Giza Egypt; ^3^ Faculty of Computer Science and Engineering New Mansoura University Mansoura Egypt; ^4^ School of Food and Biological Engineering Jiangsu University Zhenjiang China; ^5^ School of Advanced Technologies, Engineering and Science Accra Institute of Technology Accra Ghana

**Keywords:** infrared heating, modeling, onion, quality, slice thickness

## Abstract

The infrared heating process is one potential method for enhancing food quality while speeding up dehydration. We statistically evaluated slice quality based on thickness, infrared power, and airflow. This assessment focused on drying time, color, shrinkage, water activity, and rehydration ratio. The onion slices were dried with infrared powers set at 1500, 3500, and 5500 W/m^2^, airflow rates of 0.3, 0.7, and 1.0 m/s, and slice thicknesses of 4, 6, and 8 mm. We fitted the dehydrating curves from our experiments to thin‐layer drying equations. The drying times required to lower the water content to approximately 6% (wb) were between 500–430, 460–400, and 420–350 min at infrared power levels of 1500–5500 W/m^2^, respectively. Our calculations showed that the Midilli and Kucuk equation had the highest coefficient of determination (*R* ^2^) and the lowest residual sum of squares (*χ* ^2^). The results revealed that drying time increased with higher airflow and slice thickness and decreased with greater intensity. The mean activation energy was 19–30 kJ/mol across the different conditions. The shrinkage ratio varied with airflow speed, and the rehydration ratio changed in response to increasing infrared radiation intensity and airflow. Furthermore, this research underscores the potential of advanced heating technologies to improve food drying processes, offering valuable insights for the food industry as it seeks optimization efficiency.

## Graphical abstract

This assessment focused on drying time, color, shrinkage, water activity, and rehydration ratio. Onion slices were dried using infrared powers 1500, 3500, and 5500 W/m^2^, airflow rates 0.3, 0.7, and 1.0 m/s, and thicknesses 4, 6, and 8 mm. Midilli and Kucuk most effectively improved the drying process of onion slices in a thin layer. This research underscores the potential of advanced heating technologies to enhance food drying processes, providing valuable insights for the food industry seeking optimization efficiency.

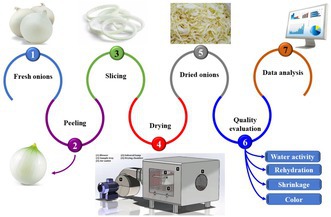

## 1 Introduction

Due to their high moisture content, agricultural products such as vegetables and fruits are classified as perishable foods (Younas et al. 2021). Free water elimination is an effective method for preserving food for off‐season usage, as it prevents enzymatic reactions and microbiological deterioration (El‐Mesery and Elabd 2021). Dehydrating is a commonly known procedure that decreases the requirement for storage intergalactic and transport mass, prevents the product from absorbing free water, and extends its shelf life. Dried fruits are optimal for making fruits more available, as they have an extended shelf life. It is also crucial to progress and increase the marketplace for best consumer‐dried foods with satisfactory color, form, and rehydration abilities (Castillo‐Téllez et al. 2017; Salehi 2020a).

The application of infrared technology for drying agricultural products is becoming increasingly prevalent, with traditional methods being supplanted in many instances. The utilization of the infrared drying method in foods has numerous benefits. The direct heating of the material facilitates the rapid drying process (Salehi 2020b). Furthermore, infrared drying requires significantly less energy than hot‐air drying. Infrared energy is a form of electromagnetic radiation located just beyond the red end of the visible light spectrum (Salehi and Satorabi 2021). It is frequently utilized for heating due to its capacity to be absorbed by the substance and transformed into thermal energy (Ye et al. 2021). A heating element engineered to facilitate infrared energy transfer is intended to emit infrared radiation. This can be accomplished using many methods, including carbon or ceramic components, which release infrared radiation upon heating. Rather than warming the surrounding air, these heating components convey heat directly to the material's surface via infrared radiation. This method enhances efficiency by circumventing the necessity to heat air, thereby allowing heat transmission to the substance through convection (Chen et al. 2019). This heating technology may improve energy efficiency by reducing heat loss to the ambient air. Heat is precisely directed to the required areas, resulting in expedited heating times and less energy use. This heating element is utilized in diverse applications, such as infrared heaters in home and commercial environments, industrial procedures necessitating direct material heating, and certain cooktops and ovens. The unheated surrounding air enhances safety and comfort in the environment. In a space heated by infrared radiation, the air temperature may be lower than conventional heating; nonetheless, objects and individuals within the room might still experience warmth due to direct heat transfer (Zhang et al. 2022).

Several empirical and semi‐empirical mathematical models have been utilized to analyze food drying. At the same time, while the thin‐layer model may be used to recreate the drying curve under identical conditions, it can also be used to estimate the mass transfer that occurs throughout the drying process (Puente‐Díaz et al. 2013). The mathematical modeling represents the optimal methodology for characterizing the kinetics of the drying procedure. The conceptualization and modeling of various mass movement operations, such as a process known as dehydration and the emission of water during preservation, can be enhanced by appropriate water diffusivity (Hu et al. 2022). The computational simulation of infrared drying of various crops, including garlic slices (El‐Mesery et al. 2022), carrots (Botelho et al. 2011), pineapples (Baptestini et al. 2016), bananas (Pekke et al. 2013), ginger (Osae et al. 2020), and tomatoes (Sadin et al. 2014), has been the subject of several studies in recent years. Despite the extensive research in this area, much is still to be learned about drying food products using infrared energy. For instance, no study has examined the impact of sample thickness and drying conditions on the quality of onion slices in an infrared heating system. The objective of this study was to evaluate the impacts of airflow, slice thickness, and infrared power on drying time, as well as physics‐quality strictures and drying kinetics.

### 1.1 Research Hypothesis

This study proposes that infrared‐convective drying parameters, which comprise infrared power, airflow velocity, and slice thickness, directly affect both the drying kinetics (drying time, moisture diffusivity) and the physicochemical quality (color, shrinkage, rehydration ratio, water activity) of onion slices. It further contends that the Midilli and Kucuk thin layer drying model will most accurately capture dehydration behavior across varied experimental conditions, with moisture diffusivity and activation energy decreasing as infrared intensity and airflow velocity increase. Moreover, the study anticipates that higher infrared power and airflow rates will improve drying efficiency while accelerating browning via Maillard reactions. In contrast, thicker slices will prolong drying time and diminish rehydration capacity, aligning directly with the objectives, variables, and outcomes presented in the abstract and conclusion, ensuring a clear, testable, and coherent framework.

## 2 Materials and Methodology

### 2.1 Materials

**FIGURE 1:**
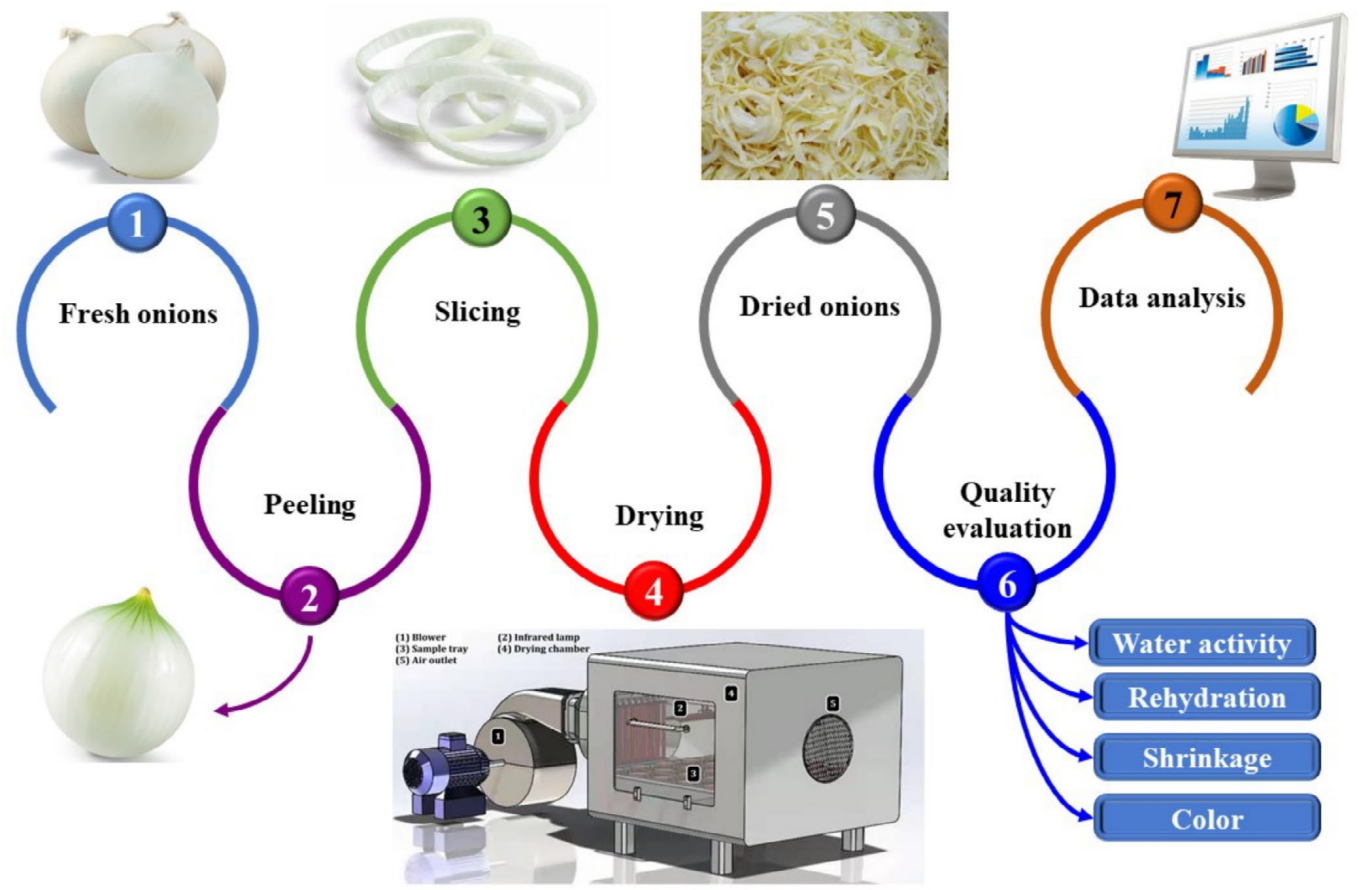
Flowchart of the drying of infrared drying white onion slices processing.

Fresh white variety onions were obtained from a local market. The onions were similar in color and ripening stage and were refrigerated at 4°C. After settling at ambient temperature for 2 h, the samples were removed from the refrigerator. The slicing machine cut the onions into different thicknesses: 4, 6, and 8 mm. Figure 1 presents the experimental flowchart.

### 2.2 Experimental Setup

**FIGURE 2:**
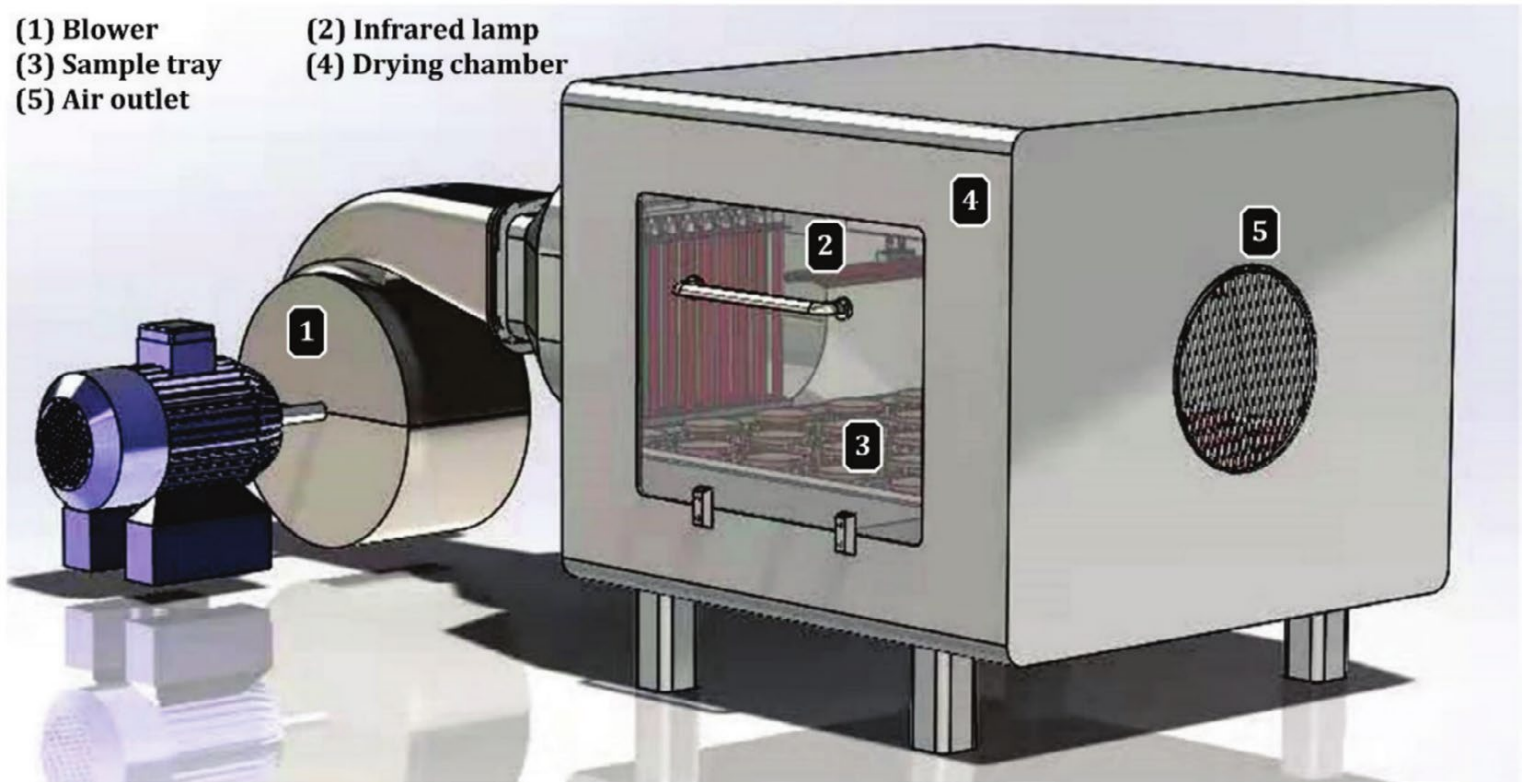
Schematic diagram of the convection‐infrared dryer.

A schematic representation of the dryer is provided in Figure 2. The apparatus includes a conveyor belt system, a drying cabinet, and both infrared and convective techniques with associated measurement capabilities. The drying space consists of chambers measuring (0.8 × 0.8 × 0.60 m). An aluminum layer coated with an infrared reflective material, 0.15 cm thick, was applied to the inner surface of the drying chamber. A 5 cm thick layer of asbestos was used as insulation for the external walls. Inside the drying chamber, 1000 W halogen infrared heater lamps with a diameter of 35.5 cm were installed. These lamps also featured a diameter of 0.6 cm. The infrared lamps with trays were aligned parallel to a stable 15 cm slit. The output power of the infrared radiation can be adjusted by regulating the voltage with the help of a power regulator. The convective unit consists of two electrical heaters and a 1.4 kW fan, which are responsible for generating the necessary drying airflow. Subsequently, the air flows through a PVC tube and enters the drying cabinet via two inlets when air flows through electric heaters with a power output of 1.5 kW, its temperature increases.

### 2.3 Drying Operation

The appliance was deactivated for 30 min to maintain uniform drying settings. Five hundred grams of onions were placed in an evenly distributed thin layer on a drier below the wire mesh tray. The onion dehydrating employed the following variables: slice widths of 4, 6, and 8 mm; airflow rates of 0.3, 0.7, and 1.0 m/s; and infrared power of 1500, 3500, and 5500 W/m^2^. The drying procedure was reiterated until the onion slices attained a final water content of about 6% ± 0.3% (wb).

### 2.4 Moisture Content Determination

Using the method outlined by AOAC (2005), a representative sample of onion slices weighing about 20 g was dried on a plate in an oven at 105°C for 24 h. This was done to control the original water content of the onion slices. A more reliable average was obtained by conducting three separate runs to generate the average. The process was repeated three times to provide a more reliable average. The initial moisture content of the samples was determined to be 85.8% ± 0.1% weight‐to‐volume. The MC of the slices, abbreviated as MCdb, was presented in Equation (1):

(1) $${\mathrm{MC}}_{\mathrm{db}}=\frac{W_{i}-W_{d}}{W_{d}}$$

*W*_*i*_ and *W*_d_ signify the sample's beginning and dried weight, respectively.

The final moisture content (*M*_f_) was determined dryly following Equation (2).

(2) $$M_{f}=\frac{W_{\mathrm{wet}}-W_{d}}{W_{d}}$$

(3) $${\mathrm{MC}}_{t}=\frac{M_{t}-M_{o}\;\left(1-{\mathrm{MC}}_{o}\right)}{M_{o}\;\left(1-{\mathrm{MC}}_{o}\right)}$$

### 2.5 Modeling of the Drying Behavior

Numerical modeling of dehydrating reactions and dynamics is a controlling mechanism for procedures determining the optimal dehydration method for a slice. The developed models facilitate the construction of new dehydrating structures, identify optimal drying constraints, and anticipate concurrent mass and heat transport phenomena throughout the drying process. Accurate modeling of drying behavior produces excellent products and improves energy efficiency. This research employed 11 semi‐empirical dehydrating models to outline the dehydrating kinetics of onions (Darvishi et al. 2014; Salehi, Inanloodoghouz, et al. 2023).

Equation (4) is operated to estimate the moisture ratio during drying.

(4) $$\mathrm{MR}=\frac{M_{t}-M_{e}}{M_{i}-M_{e}}$$

Because the equilibrium moisture content (*M*_e_) is insignificant, the moisture ratio can be calculated using Equation (5) [Li et al. 2020; Mayor and Sereno 2004].

(5) $$\mathrm{MR}=\frac{M}{M_{i}}$$

Equation (6) was used to compute the (Dr) drying rate [25].

(6) $$\mathrm{Dr}=\frac{M_{t+\mathrm{dt}}-M_{t}}{\mathrm{dt}}$$

A non‐linear numeric analysis was carried out to incorporate the numerical information into 11 semi‐theoretical thin‐layer equations.

*Newton model*

(7) $$\mathrm{MR}=\exp\left(-\mathrm{kt}\right)$$

*Page model*

(8) $$\mathrm{MR}=\exp\left(-kt^{n}\right)$$

*Henderson and Pabis model*

(9) $$\mathrm{MR}=a.\exp\left(-\mathrm{kt}\right)$$

*Modified Henderson and Pabis model*

(10) $$\mathrm{MR}=a.\exp\left(-\mathrm{kt}\right)+b.\exp\left(-\mathrm{gt}\right)+a.\exp\left(-\mathrm{ht}\right)$$

*Logarithmic model*

(11) $$\mathrm{MR}=a.\exp\left(-\mathrm{kt}\right)+C$$

*Wang and Singh model*

(13) $$\mathrm{MR}=1+\mathrm{at}+bt^{2}$$

*Verma et al. model*

(14) $$\mathrm{MR}=a.\exp\left(-\mathrm{kt}\right)+\left(1-a\right)\exp\left(-\mathrm{gt}\right)$$

*Thomson model*

(15) $$t=a.\ln\mathrm{MR}+b.{\left[\ln\left(\mathrm{MR}\right)\right]}^{2}$$

*Two‐term model*

(16) $$\mathrm{MR}=a.\exp\left(-k_{o}t\right)+b.\exp\left(-k_{1}t\right)$$

*Modified Page model*

(17) $$\mathrm{MR}=\exp\left[{\left(-\mathrm{kt}\right)}^{n}\right]$$

*Midilli and Kucuk model*

(18) $$\mathrm{MR}=a.\exp\left(-kt^{n}\right)+b.t$$

### 2.6 Moisture Effective Diffusion

The drying of food products is achieved by interior diffusion, typically during the time‐decreasing rate. Fick's second rule has been the basis for developing several mathematical equations published to depict drying processes throughout the decreasing rate phase. To demonstrate, use Equation (19).

(19) $$\frac{\partial \;M}{\partial \;t}=D_{\mathrm{eff}}\frac{\delta^{2}M}{\delta x^{2}}$$

Diffusivity is measured in (m^2^/s) *δM*/*δt*, moisture content in db/s, and *x* thickness in m.

Based on the hypotheses listed below, it is possible to arrive at the mathematical Equations ((20), (21), (22), (23), (24), (25)) by assuming that the geometry is that of a slab with moisture equally distributed at a concentration *M*_o_ and all diffusion taking place only in the *x* direction.

#### 2.6.1 Assumptions

- A sample's bulk initially has a uniform distribution of moisture.
- When the center is considered, mass transfer is symmetric.
- The sample's surface moisture content instantly equates with the air quality.
- Surface resistance to mass transfer is negligible compared to the sample interior resistance.
- Mass transfer is only by diffusion.
- The Diffusion coefficient is stable, and the shrinkage is minimal.

*Initial conditions (t = 0)*

(20) $$M=M_{o}\;0\leq X<L$$

*Boundary conditions (t > 0)*

(21) $${\left.\frac{\mathrm{\delta M}}{\mathrm{\delta X}}\right|}_{x=0}=0$$

(22) M=0X=L

(23) $$\mathrm{MR}=\frac{M}{M_{i}}=\frac{8}{\pi^{2}}\;\sum_{n=0}^{\infty }\frac{1}{{\left(2n-1\right)}^{2}}\exp\left[-{\left(2n-1\right)}^{2}\;\pi^{2}\;\frac{D_{\mathrm{eff}}}{4L^{2}}\;t\right]$$

*D*_eff_ is the effective moisture diffusivity in m^2^/s, *t* is the drying time in seconds, MR is the moisture ratio, and *L* is half the sample thickness (m).

(24) $$\mathrm{MR}=\frac{8}{\pi^{2}}\exp\left[-\frac{\pi^{2}D_{\mathrm{eff}}\;t}{4L^{2}}\right]$$

Taking the logarithm on both sides of Equation (24) results in Equation (25).

(25) $$\ln\left(\mathrm{MR}\right)=\ln\left(\frac{8}{\pi^{2}}\right)-\left(\pi^{2}\frac{D_{\mathrm{eff}}}{4L^{2}}t\right)$$

The slopes approach is utilized to calculate the diffusion coefficient. The slope of the plot of ln MR versus time at various temperatures is used to calculate the effective moisture diffusivity, or “*D*_eff_.”

The slope of the plot of ln MR versus time at various temperatures is used to calculate the “*D*_eff_,” according to Equation (26).

(26) $$\text{Slope}=\frac{\pi^{2}D_{\mathrm{eff}}}{4L^{2}}$$

### 2.7 Activation Energy

Equation (27) shows how the energy of activation (*E*_a_) was computed using an Arrhenius‐type equation (Doymaz 2012).

(27) $$D_{\mathrm{eff}}=D_{o}\exp\left(-\frac{E_{a}}{R\;T}\right)$$

where *E*_a_ is the activation energy (kJ/mol), *R* is the universal constant of gas (kJ/mol), *T* is the standard air temperature (*K*), and *D*_o_ is the Arrhenius equation's pre‐exponential factor (m^2^/s). The energy required for activation was estimated by dividing the slope of the Arrhenius plot by 1/*T*.

(28) $$\ln\left(D_{\mathrm{eff}}\right)=\ln\left(D_{o}\right)-\left(\frac{E_{a}}{R\;T}\right)$$

A plot of ln *D*_o_ versus 1/*T* from Equation (28), with a slope (*K*) from Equation (29).

(29) $$K=\frac{E_{a}}{R}$$

### 2.8 Water Activity

The samples (3 g) were subjected to a water activity (*a*_w_) assessment at 25°C ± 0.1°C. The apparatus employs a resistive, electrolytic sensor to quantify the moisture content of the surrounding air within a regulated chamber, thereby facilitating an accurate and dependable assessment of the water activity (*a*_w_).

### 2.9 Color Measurement

The material was subjected to five color tests. The total color alteration and browning index (BI) in the fresh item was calculated utilizing Equations (30) and (32) (Zeng et al. 2019):

(30) $$\mathrm{\delta E}=\sqrt{{\left(L^{*}-L_{o}^{*}\right)}^{2}+{\left(a^{*}-a_{o}^{*}\right)}^{2}+{\left(b^{*}-b_{o}^{*}\right)}^{2}}$$

(31) $$\mathrm{BI}=100\times \left(\frac{x-0.31}{0.17}\;\right)$$

(32) $$X=\frac{a^{*}+1.75\;L^{*}}{\left(6.645\;L^{*}+a^{*}-3.012\;b^{*}\right)}$$

### 2.10 Shrinkage Ratio

Different dehydration techniques notice nutrition shrinkage as a typical chemical reaction. Accurate evaluation of the water and temperature profiles in hydrated objects is essential, as these variations impact the outcome. Subsequently, the mean principles were reported (Velić et al. 2007). The shrinkage ratio (*S*_r_) was determined using Equation (33).

(33) $$S_{r}=1-\frac{V_{d}}{V_{o}}$$

*V*_o_ and *V*_d_ denote the mean volume of onion slices before and after drying.

### 2.11 Rehydration Ratio

The present study introduced 150 mL of purified water into a 500 mL beaker. Subsequently, the glass was closed and heated for 5 min. After measuring the samples, the rehydration ratio (*R*_r_) was calculated using Equation (34).

(34) $$R_{r}=\frac{M_{r}}{M_{d}}$$

where *M*_d_ and *M*_r_ are the mass of dehydrated and rehydrated onion slices, respectively.

### 2.12 Model Performances

The experimental data are presented as means ± standard deviations (SD). Statistical analyses were performed using SPSS Statistics software. The effects of various operating settings on the drying properties and quality parameters were assessed using an analysis of variance (ANOVA), followed by a post hoc Duncan's multiple range test at a significance level of 0.05. The equation with the lowest RMSE, *χ* ^2^, and maximum *R* ^2^ values was optimal, as it most clearly defined the drying kinetics (El‐Mesery 2022; Ozyalcin et al. 2023).

(35) $$R^{2}=\frac{\sum_{i=1}^{N}{\left({\mathrm{MR}}_{\exp.i}-{\mathrm{MR}}_{\mathrm{Pre}.i}\right)}^{2}}{\sqrt{\left[\sum_{i=1}^{N}{\left({\mathrm{MR}}_{\exp.i}-{\mathrm{MR}}_{\mathrm{Pre}.i}\right)}^{2}\right]*\left[\sum_{i=1}^{N}{\left({\mathrm{MR}}_{\exp.i}-{\mathrm{MR}}_{\mathrm{Pre}.i}\right)}^{2}\right]}}$$

(36) $$\text{RMSE}=\sqrt{\;\frac{\sum_{i=1}^{N}{\left({\mathrm{MR}}_{\exp.i}-{\mathrm{MR}}_{\mathrm{Pre}.i}\right)}^{2}}{N}}$$

(37) $$\chi^{2}=\frac{\sum_{i=1}^{N}{\left({\mathrm{MR}}_{\exp.i}-{\mathrm{MR}}_{\mathrm{Pre}.i}\right)}^{2}}{N-n}$$

## 3 Results and Discussion

### 3.1 Drying Time

**FIGURE 3:**
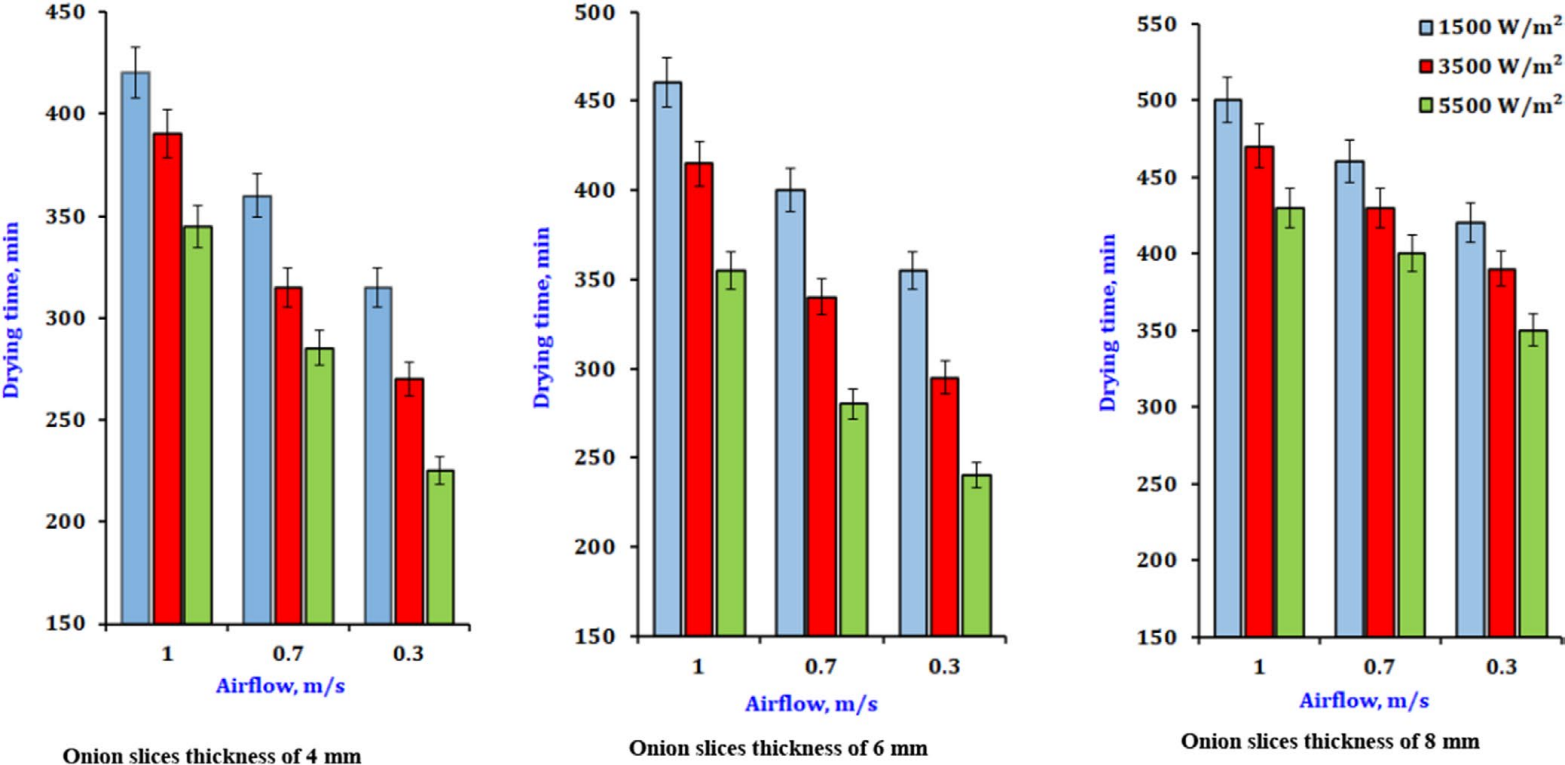
The effect of drying conditions on drying time for dry onion slices at different slice thicknesses.

Figure 3 illustrates the impact of various drying parameters on the duration of the infrared dehydrating for different slice thicknesses, considering multiple levels of infrared power and airflow. The drying durations required to reduce the water content to about 6% (wb) were 500–430, 460–400, and 420–350 min at infrared power ranging from 1500 to 5500 W/m^2^, respectively. Increased infrared power will result in higher product temperatures and heat absorption, enhanced water transmission pushing force, accelerated dehydrating rate, and reduced drying duration. The rise in air velocity, while maintaining a constant level, resulted in an extended drying time attributed to the intensity of infrared radiation. The data indicate a decrease in the product's drying rate with increased air velocity. This pattern was applicable at all levels within the parameters of the current study. The item in question will cool, leading to a decrease in dripping airflow. The optimal combination of intensity and airflow rate may lead to a reduction in drying duration. The falling rate period indicates that the initial weight loss resulted from the vaporization of unbound surface moisture content (MC), while the weight loss following this period was due to bound moisture (El‐Mesery et al. 2024). Similar patterns of increased weight loss at higher times and temperatures were noted in drying onion and garlic slices (Demiray and Tulek 2017; El‐Mesery et al. 2022). In the initial stages, drying rates driven by heat and mass transfer were high, and moisture was rapidly removed because of the significant temperature difference between the hot air oven and the product's surface.

As shown below in Equation (38), the drying time could be calculated within the parameters of this work, as shown in Figure 3, for any recorded airflow (*V*) and value of intensity (*I*).

(38) $$\text{Drying time}=\frac{45.3+40\;V-15.3\;\ln I}{1+0.01\;V+0.95\;\ln I+0.21\;{\left(\ln I\right)}^{2}}\;R^{2}=0.989$$

**FIGURE 4:**
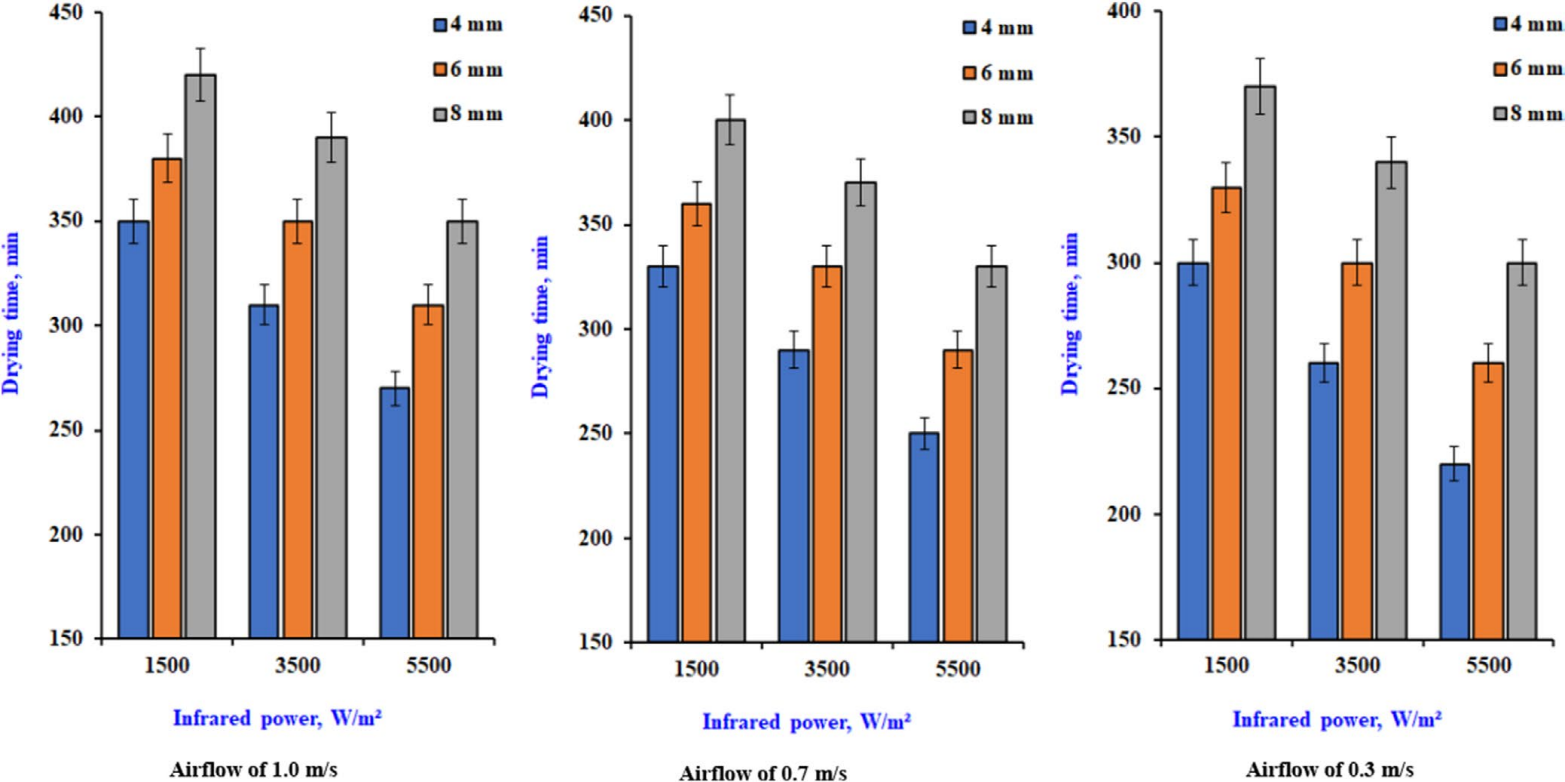
The effect of drying conditions on drying time for dry onion slices at different drying conditions.

Figure 4 demonstrates how different slice dimensions affect the length of the drying procedure. The examination is performed under constant airflow and infrared radiation levels of 0.3 m/s and 0.55 W/cm^2^, respectively. The drying frequency increased by 80% and 188% as the slice thickness rose from 4 to 8 mm, respectively. The drying period increased with thickness, as a thicker slice retains more moisture while maintaining an equal outside area exposed to infrared power. Moreover, drying a thicker slice presents more significant challenges due to the extended diffusion pathway for moisture to exit from the interior. A prior study by Sacilik and Elicin (2006) revealed propensities similar to those in this report.

As shown below in Equation (39), the drying time could be calculated within the parameters of this work for any recorded slice thickness (Th) and intensity value (*I*).

(39) $$\text{Drying time}=\frac{75.094+81.86\;\mathrm{Th}-5.98\;T^{2}-154.93\;I}{1-0.09\;\mathrm{Th}+1.01\;I+0.65\;I^{2}}\;R^{2}=0.998$$

### 3.2 Computational Modeling Simulation of Drying Curves

**FIGURE 5:**
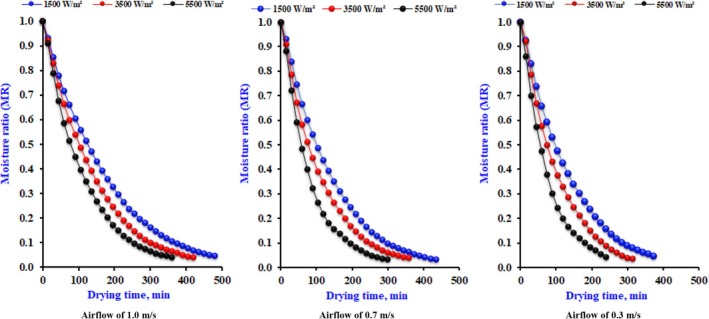
The observed data dynamics for moisture ratio for onion slices 4 mm under different infrared intensity and airflow.

**FIGURE 6:**
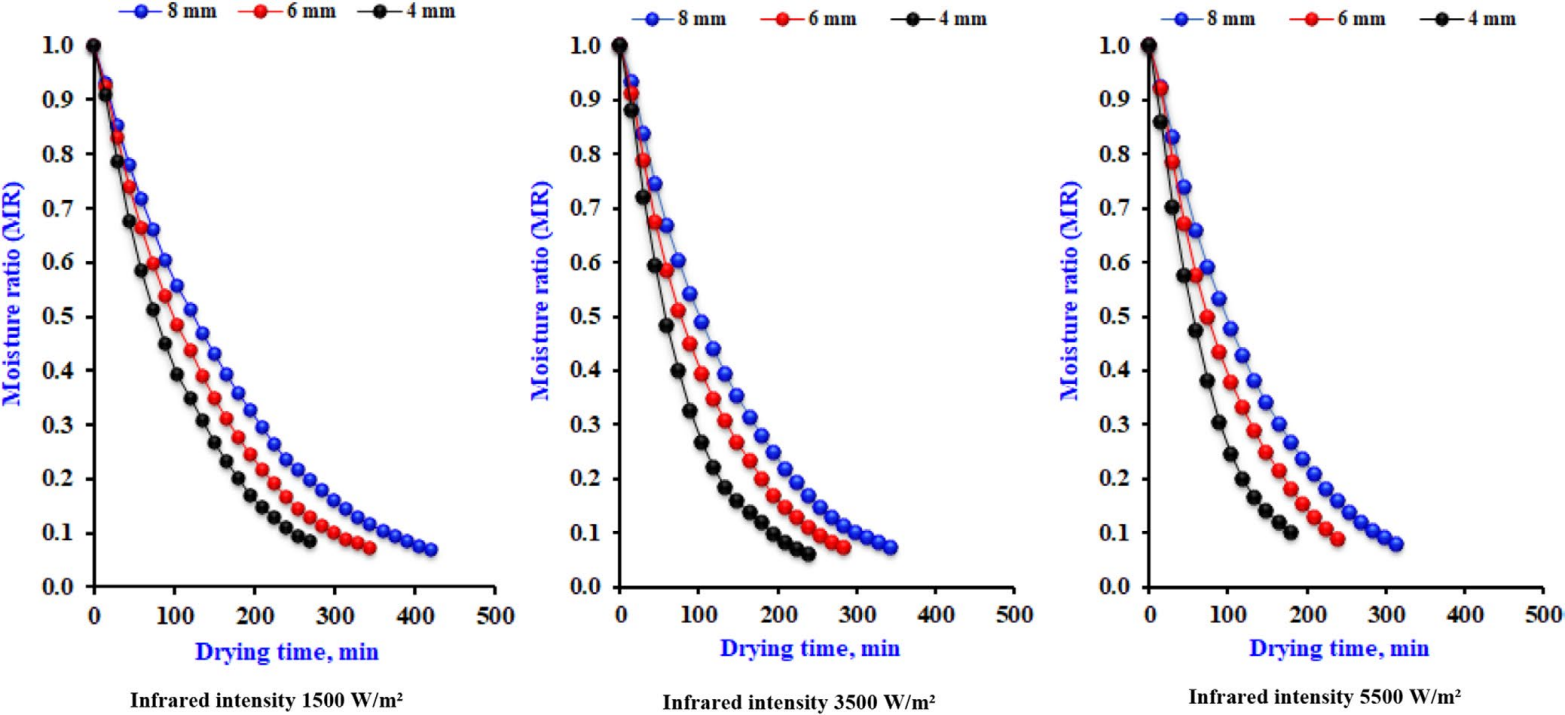
The observed data dynamics for moisture ratio for different onion slices thickness under different infrared intensity.

Figure 5 illustrates the impact of various drying parameters on the MR of the infrared dehydrating for different slice thicknesses, considering multiple levels of infrared power and airflow. The average outcomes of the statistics regarding how much data conforms to various dehydrating models are reported. All equations accurately predicted drying behavior. This indicates that each model can effectively suggest the infrared dehydrating process of onions. The Midili and Kucuk model demonstrated the most muscular fit, evidenced by the most outstanding *R* ^2^ values and the lowest *χ* ^2^ and RMSE (Table 1). After the statistical analysis, the constants from each model were employed to evaluate the MR for each specified value. Figure 6 compares the assessed MR and the determined value across different infrared radiation intensities, maintaining a constant air velocity of 1.0 m/s.

**TABLE 1:** Statistical variables obtained from the fitting of various thin‐layer drying models to experimental moisture ratio during drying of onion slices.

Name of model	Statistical parameters							
	*R* ^2^	SE	*χ* ^2^	RMSE	EF	SSE	SEE	SD
Newton	0.99845	0.00974	0.000191	0.01320	0.99780	0.00018	0.01353	0.0134
Henderson and Pabis	0.99803	0.01090	0.000137	0.01116	0.99841	0.00013	0.01144	0.0111
Page	0.99869	0.00772	0.000065	0.00758	0.99923	0.00006	0.00777	0.0076
Modified Page	0.98862	0.02547	0.011504	0.08484	0.87375	0.01081	0.08729	0.0347
Logarithmic	0.99881	0.00755	0.000067	0.00790	0.99862	0.00046	0.00735	0.0080
Two‐term	0.99869	0.00704	0.000069	0.00702	0.99897	0.00016	0.00791	0.0076
Modified Henderson and Pabis	0.99895	0.00852	0.000070	0.01196	0.99853	0.00038	0.00741	0.0077
Midilli et al.	0.99973	0.00500	0.000049	0.00593	0.99965	0.00006	0.00582	0.0057
Verma et al.	0.99876	0.00788	0.000083	0.00831	0.99904	0.00007	0.00853	0.0084
Wang and Sing	0.99062	0.02962	0.001084	0.03133	0.98733	0.00103	0.03211	0.0314
Thompson	0.94046	0.05069	0.014658	0.11802	0.82997	0.01395	0.12096	0.1093

Additionally, at reduced radiation intensities, the expected sites exhibit a lower degree of distribution. The “Midilli and Kucuk” was modified to accommodate statistics collected at different levels of infrared and airflow, reflecting its relevance. Table 2 presents the drying constants (*k*) and *b*, the model coefficients (*a*) and *n*, and the statistical variables related to the fit quality. The magnitudes of the model parameters and coefficients are similar to those obtained by other researchers (Singh and Kumar 2012). The (*k*) increases with higher infrared levels and decreases with drying airflow. The coefficients of *a*, *n*, *b*, and constant *k* (min^−1^) were utilized in a multiple regression versus radiation (*I*) and drying air velocity (*V*) in m/s to account for the effect of drying variables on the Midilli and Kucuk model. The equations expressing these correlations take the form of Equations ((40), (41), (42), (43)).

(40) $$k=0.017+0.14\;I-0.045\;V-0.29\;I^{2}+0.016\;V^{2}+0.034\;I.V\;R^{2}=0.858$$

(41) $$a=1.11-3.23\;I+0.39\;V+6.76\;I^{2}-0.015\;V^{2}+0.026\;I.V\;R^{2}=0.847$$

(42) $$n=1.035-0.37\;I+0.11\;V+1.02\;I^{2}-0.045\;V^{2}-0.159\;I.V\;R^{2}=0.829$$

(43) $$b=0.0013-0.06\;I+0.0073\;V+0.12\;I^{2}-0.003\;V^{2}-0.0023\;I.V\;R^{2}=0.879$$

**TABLE 2:** Statistical results of Midilli et al. model and its constants and coefficients at different drying conditions.

IR (W/m^2^)	*V* (m/s)	Constants				Statistical parameters							
		*k*	*a*	*n*	*b*	*R* ^2^	SE	*χ* ^2^	RMSE	EF	SSE	SEE	SD
1500	1.5	0.008	0.999	1.0580	0.00004	0.99976	0.0045	0.00002	0.0021	0.9997	0.00007	0.0032	0.0032
	1	0.001	1.007	1.0290	0.00001	0.99983	0.0063	0.00003	0.0041	0.9995	0.00004	0.0063	0.0043
	0.5	0.015	1.012	1.0440	0.00009	0.99913	0.0057	0.00007	0.0080	0.9991	0.00009	0.0092	0.0072
3500	1.5	0.010	0.999	1.0240	0.00016	0.99979	0.0042	0.00002	0.0024	0.9998	0.00000	0.0025	0.0025
	1	0.014	1.007	1.0020	0.00011	0.9996	0.0059	0.00003	0.0055	0.9996	0.00003	0.0057	0.0037
	0.5	0.019	0.783	1.0570	0.00400	0.99992	0.0043	0.00390	0.0093	0.9998	0.00008	0.0096	0.0096
5500	1.5	0.015	1.005	0.9910	0.00019	0.9998	0.0051	0.00003	0.0036	0.9997	0.00002	0.0048	0.0048
	1	0.014	1.003	1.0960	0.00005	0.99987	0.0035	0.00001	0.0102	0.9998	0.00010	0.0010	0.0080
	0.5	0.015	0.998	1.0126	0.00003	0.99999	0.0047	0.00002	0.0090	0.9997	0.00009	0.0093	0.0093

**FIGURE 7:**
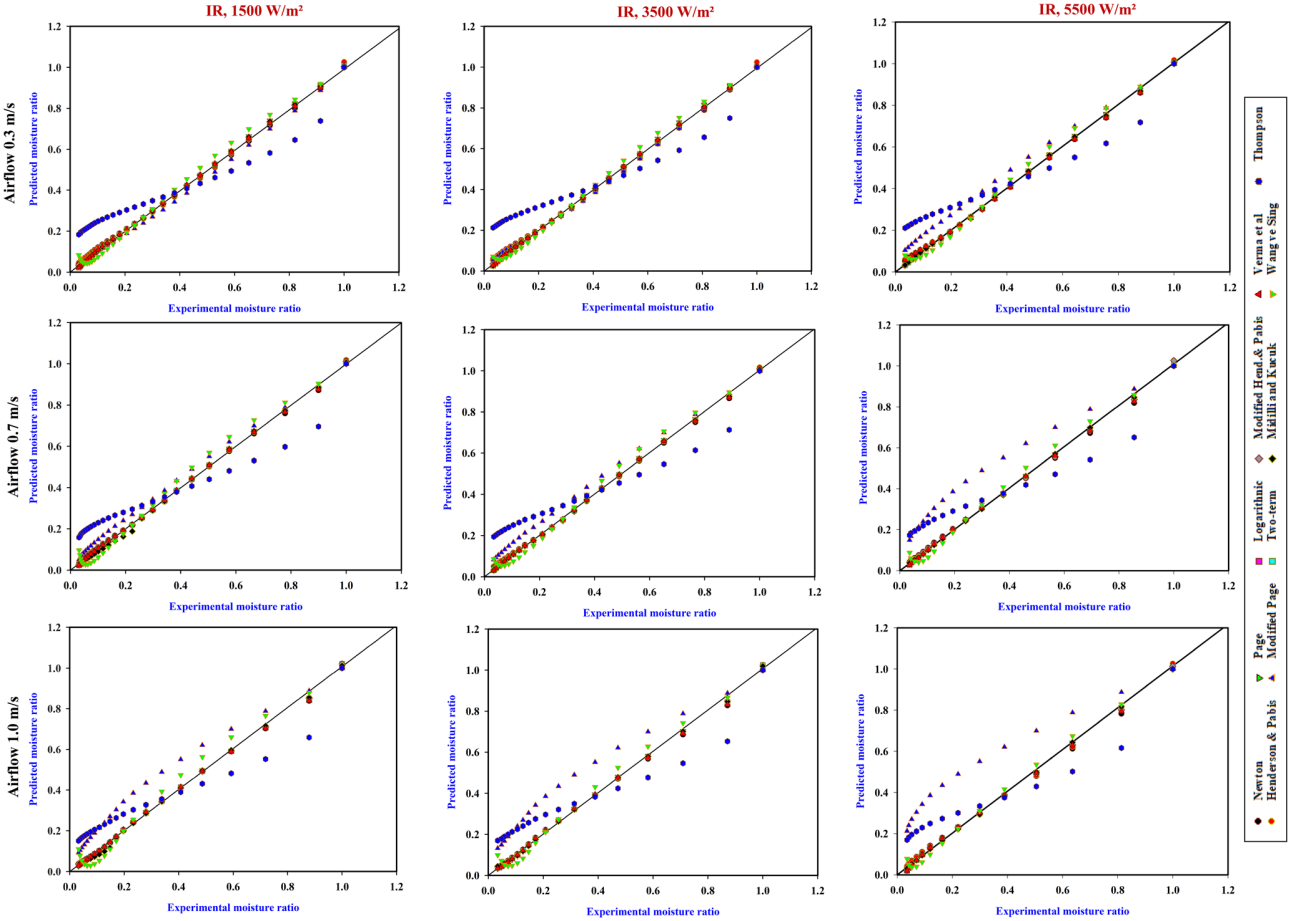
Model prediction through computational simulation and experimental moisture ratio data.

Figure 7 illustrates the predicted MR compared to data from experiments across different drying conditions. The symbols have precise alignment along a 45° straight line, demonstrating significant relationships within calculated and experimental information. The Midilli and Kucuk effectively represent the dehydrating performance of onion under various airflow and power (Sonmete et al. 2017).

### 3.3 Effective Moisture Diffusivity

Table 3 shows the effective moisture diffusivity calculated using Fick's second diffusion rule. The *D*_eff_ was estimated by operating the slopes method. The optimum moisture diffusivity results for drying slices are 5.05, 6.11, and 3.65 × 10^−10^ at 1500, 3500, and 5500 W/m^2^ under 0.3 m/s, respectively. The results showed that moisture diffusivity, rising slice thickness, and infrared intensity improved. The average energy for a vapor's transitional, rotational, and vibrational motions increases with increasing intensity, causing a more significant moisture gradient, a higher mass transfer rate, and increased moisture diffusivity. The optimum moisture diffusivity decreased with the airflow going from 0.3 to 1.0 m/s, as shown in Table 3. This could be because the higher airflow reduced the samples' temperatures, as their temperature was higher than the airflow. Reducing the supplied energy to the drying chamber consequently decreased the diffusivity of onion slices during the drying process. This conclusion aligns with the results from Hadibi et al. (2023) regarding the effect of varying drying temperatures on the effective moisture diffusivity of fruits. Furthermore, the practical moisture diffusivity values obtained were lower than 8.55 × 10^−9^ m^2^/s at 60°C when using a convective dryer for onion slice, and measured 8.11 × 10^−11^ and 1.22 × 10^−10^ m^2^/s at 60°C and 70°C, respectively, for onion using the convective dryer (Beigi et al. 2022). Finally, providing a more sensible thermal storage medium is recommended to enhance the diffusional stage of the intermittent onion drying operation.

**TABLE 3:** Effective diffusion coefficient of onion slices under different experimental conditions.

Infrared power, W/m^2^	Airflow, m/s	Slice thickness, mm	*D*_eff_, ×10^−10^, m^2^/s	*E*_a_, kJ/mol	*R* ^2^
1500	0.3	4	5.46	23.54	0.998
3500			7.54	20.01	0.995
5500			9.59	17.74	0.996
1500	0.7		4.05	25.81	0.997
3500			5.84	21.76	0.989
5500			8.09	19.02	0.991
1500	1.0		2.59	28.05	0.998
3500			3.31	23.19	0.995
5500			6.01	20.93	0.997
1500	0.3	4	5.46	23.54	0.996
3500		6	7.35	27.18	0.995
5500		8	9.07	29.83	0.998

### 3.4 Activation Energy for Drying

Table 3 shows the activation energy at all thicknesses examined. The (*E*_a_) ranged between 18 and 28.95 kJ/mol under various experimental conditions, which is within the acceptable range of activation energy for the majority of goods (12.7–110 kJ/mol) (Tuly et al. 2023). At every level of infrared, activation energy values rose as airflow surged between 0.5 and 1.5 m/s. During infrared drying, the dehydrating rate dropped as the airflow grew between 0.3 and 1.0 m/s. The measurements of (*E*_a_) of a process drop as the mean energy of the molecules increases (Lewis‐Atwell et al. 2022, Table 4).

**TABLE 4:** The impact of varying infrared intensities, air velocity, and slice thickness on fresh and dried onion slices' water activity (*a*_w_).

Run	*I*, W/m^2^	*V*, m/s	Th, mm	*a*_w_
Fresh				0.962
1	1500	0.3	4	0.412
2	3500	0.3	4	0.401
3	5500	0.3	4	0.371
4	1500	0.7	4	0.43
5	3500	0.7	4	0.410
6	5500	0.7	4	0.385
7	1500	1.5	4	0.450
8	3500	1.0	4	0.431
9	3500	1.5	4	0.391
10	3500	0.3	4	0.371
11	3500	0.3	6	0.392
12	3500	0.3	6	0.416

### 3.5 Water Activity

**FIGURE 8:**
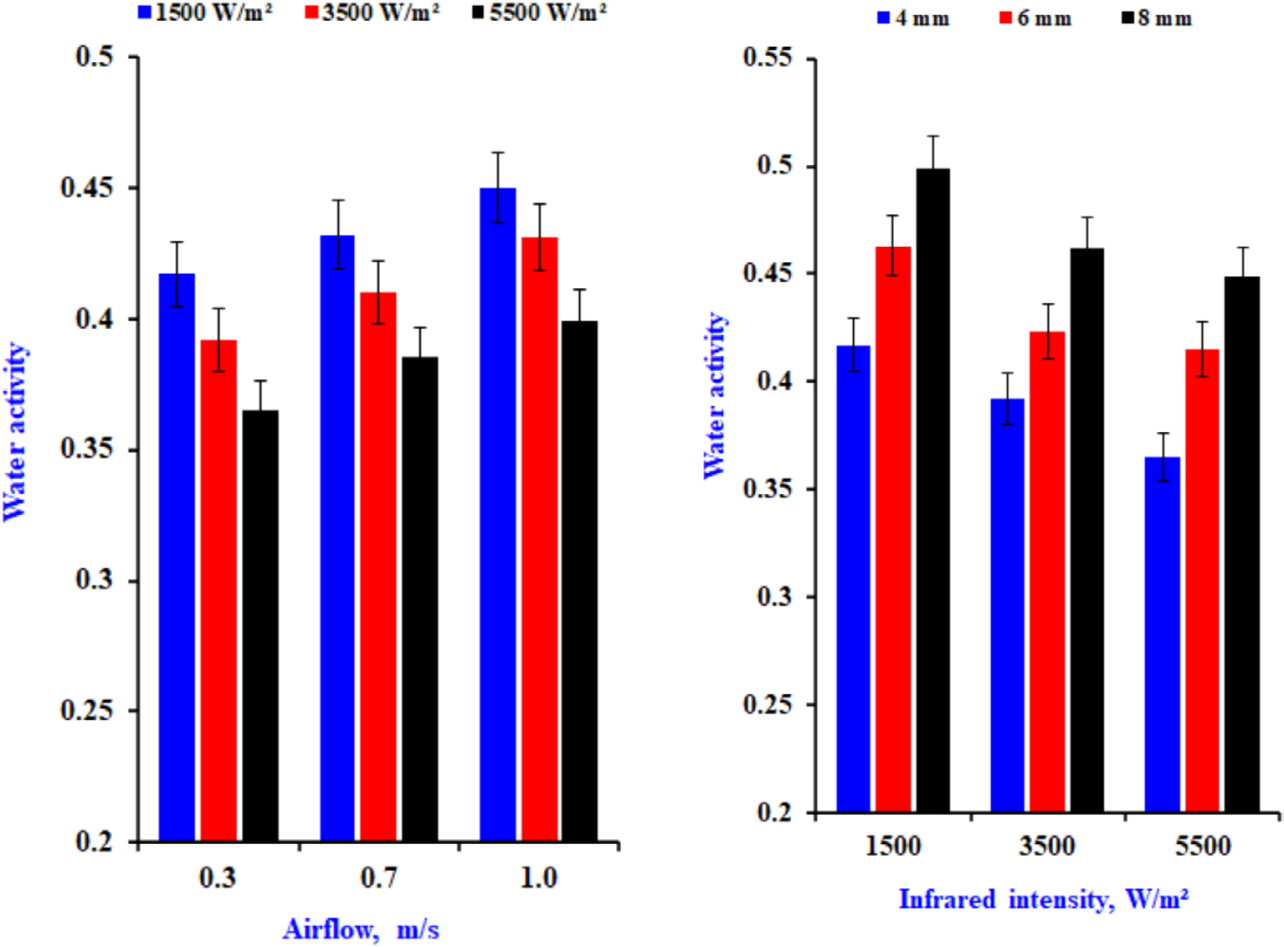
The impact of drying conditions on water activity of dry onion slices at different drying conditions.

Figure 8 displays the impact of different drying settings on the (*a*_w_) of the samples. According to all drying requirements, the (*a*_w_) slices remain constantly below 0.6, indicating that the samples are not deteriorating due to microbial activity. The average of the samples ranged from 0.38 to 0.47. The increased infrared and reduced airflow enhance water disappearance from the exteriors of the onion. The improved water loss from the superficial slice and increased water diffusion within the slices facilitate more rapid attainment of the desired water activity (Royen et al. 2020). The results of our study demonstrate that infrared drying techniques can effectively attain the desired water activity (*a*_w_) appropriate for prolonged storage durations. Various authors reported that the *a*_w_ of dehydrated minced onion was < 0.6. Most microbial counts are reduced at this *a*_w_; however, spore‐forming microorganisms may enter sporulation for sustenance. These spores could activate upon rehydration and cause spoilage through proliferation (Gomathi Padma Priya et al. 2024).

### 3.6 Color Attributes

**FIGURE 9:**
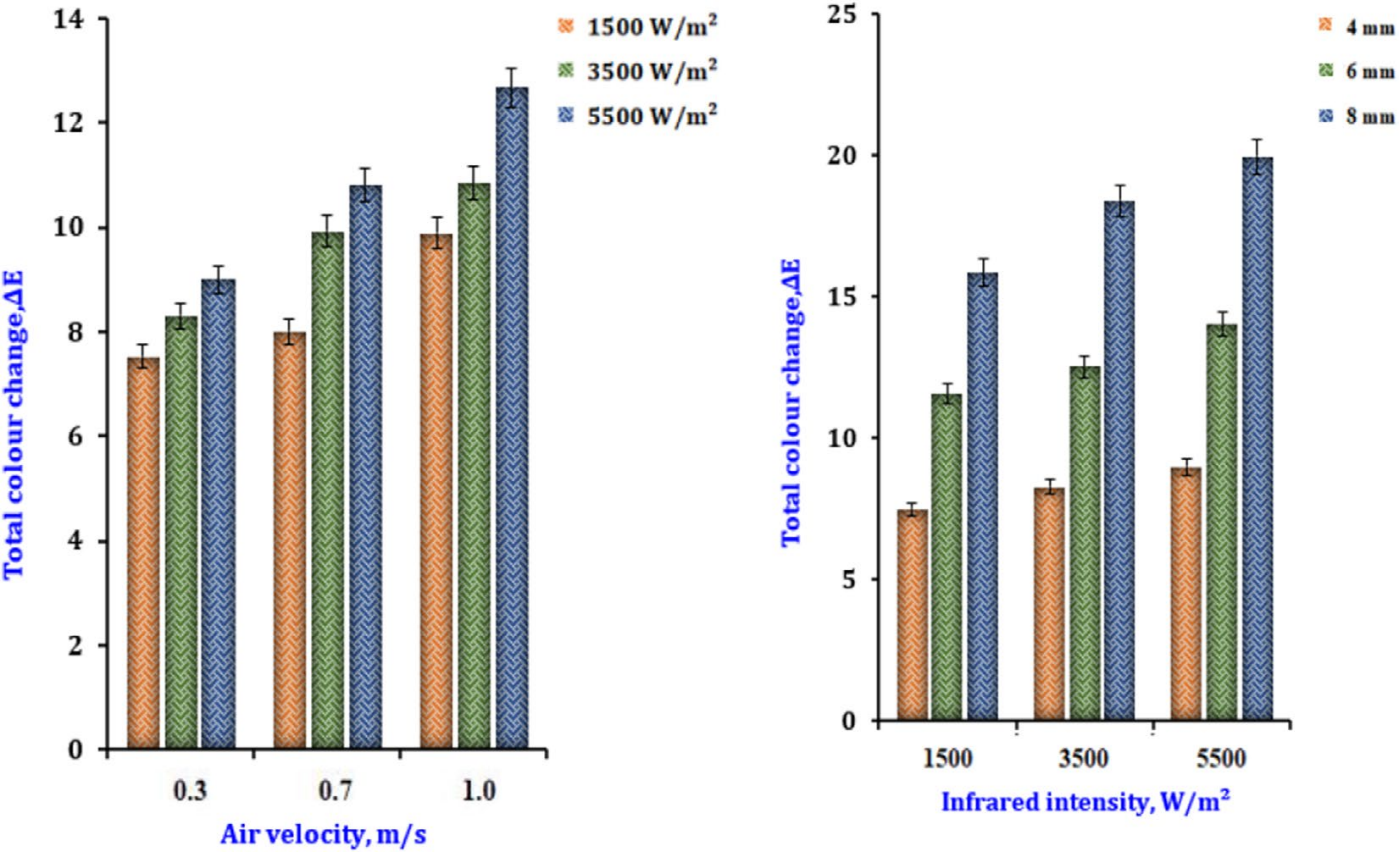
Color difference change of dried onion at different radiation intensities and slice thickness during infrared drying.

Figure 9 shows the outcome of drying settings on total color changes. The color variations rose when the airflow was 0.3 m/s, and the IR ranged from 1500 to 5500 W/m^2^. The same radiation power range was applied, and a fixed drying air velocity of 1.0 m/s increased color variation. The color change is accompanied by increased airflow and IR (Rajoriya et al. 2020).

The variations in color difference (*E*) as a function of (*V*) and (*I*) are shown in Equation (44).

(44) $$\mathrm{\delta E}=\frac{10.05-6.1\;V+0.940\;V^{2}+2.8\ln I}{1-0.92\;V+0.25\;V^{2}+0.08\ln I-0.071{\left(\ln I\right)}^{2}}\;\;R^{2}=0.999$$

An illustration of the color difference that occurs across dried slices of varying thicknesses may be seen in Figure 9. While 0.3 m/s and the power were attuned to 1500 W/m^2^, the color changes of dried samples with slices 4, 6, or 8 mm thick were 7.65, 12.47, and 19. With increasing slice thickness, the vast difference became more pronounced. The rise in *E* that occurs with growing slice thickness may be due to a prolonged drying time. This is because larger onion slices undergo a more extended oxidation period. The color difference became more pronounced as the thickness of the onion slices grew (Salehi, Goharpour, et al. 2023).

The variations in color difference (*E*) as an occupation of (*I*) and (Th) are shown in Equation (45).

(45) $$\mathrm{\delta E}=\frac{5.13-0.82\;\mathrm{Th}+7.01\;I}{1-0.31\;\mathrm{Th}+0.019\;{\mathrm{Th}}^{2}+0.302\;I}\;\;R^{2}=0.999$$

### 3.7 Browning Index

Non‐enzymatic browning serves as an additional quality indicator during the drying process. Browning is advantageous in certain processed foods but unfavorable in dried onion. The degree of browning is primarily due to color changes induced by Maillard reactions in onions. Infrared drying exhibited a lower BI value in the dried samples than in the fresh sample, attributed to pigment decomposition occurring during prolonged drying (Wu et al. 2021). Conversely, reduced *L* values were noted in other dried samples, resulting in elevated BI values. The findings indicate that integrating infrared and microwave drying markedly facilitated the browning reaction, resulting in a product with a darker hue (Zeng et al. 2023). The brightness index (BI) rises with an increase in infrared power. This trend was similarly noted in the dried onion slices (Kumar et al. 2005). Onion samples were subjected to drying in an aerobic environment using infrared methods. The elevated sample temperature enhanced the Maillard reaction and ascorbic acid oxidation, forming brown‐colored substances (Jiang et al. 2017). At an infrared power level of 5500 W/m^2^, an increase in airflow resulted in a higher BI value. The elevated temperature of infrared power facilitated the browning reaction and starch gelatinization (Li et al. 2020). The BI value of the dried sample was influenced by both the sample temperature and the drying duration. In infrared drying, a thicker sample generally leads to a higher browning index, meaning more browning occurs when compared to a thinner sample, as the heat penetration is less efficient and can cause more Maillard reactions within the thicker material, resulting in a darker color change.

### 3.8 Shrinkage Ratio

**FIGURE 10:**
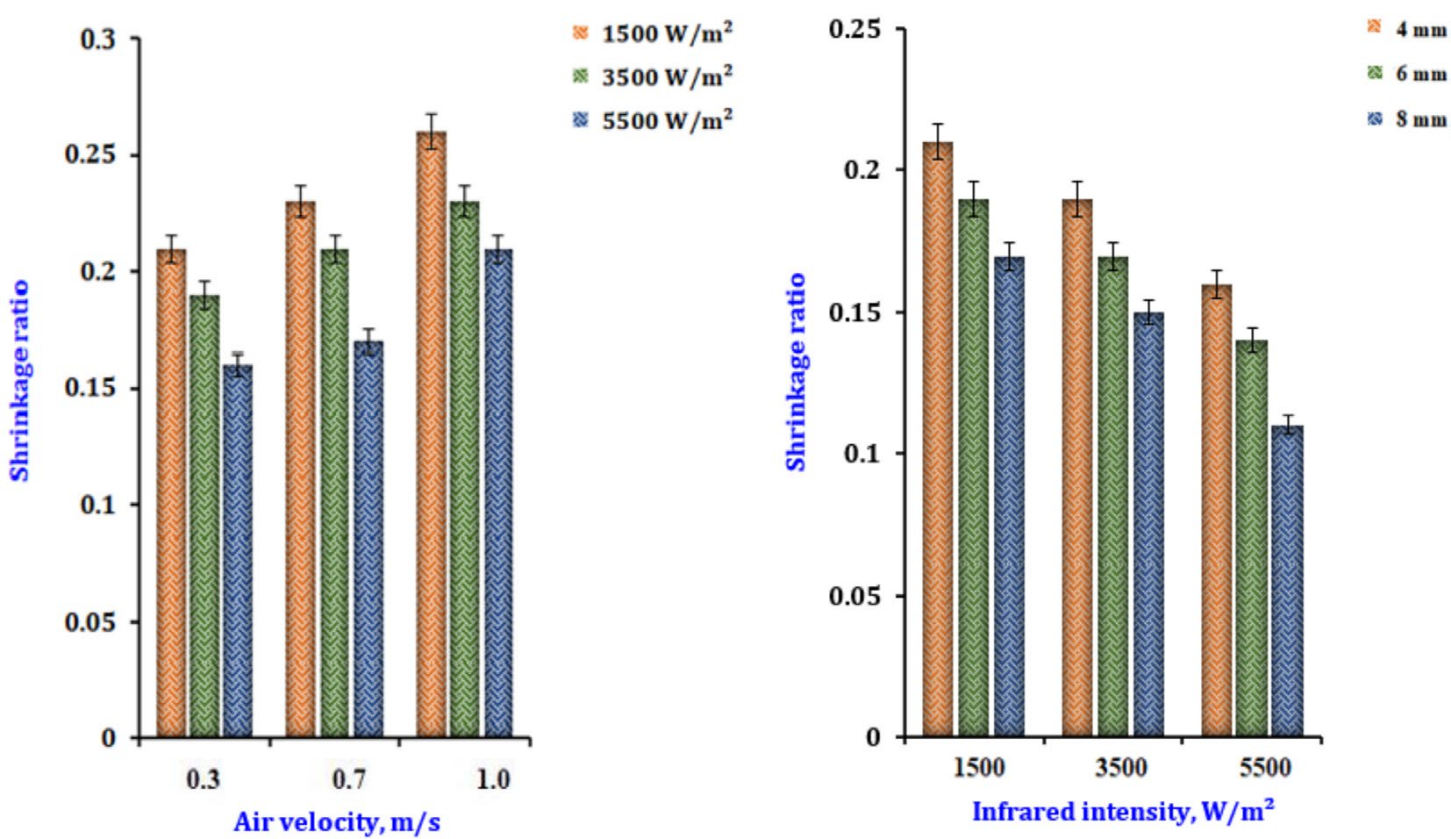
The effect of infrared radiation intensity and slice thickness on the shrinkage ratio of dried onion.

The (*S*_r_) samples dehydrated through infrared are shown in Figure 10. With the power increased from 1500 to 5500 W/m^2^, the (*S*_r_) released decreased from 0.25 to 0.16, and the airflow was maintained at 0.3 m/s. The *S*_r_ decreased from 0.25 to 0.21 while maintaining the same IR power and airflow of 1.0 m/s. Therefore, the lowest values of the shrinkage ratio were obtained by operating at a low airflow and a considerable infrared radiation intensity of 1500 W/m^2^. Under increasing intensity and low airflow conditions, a small amount of shrinkage occurs on both sides of the onion's outer surfaces. This is because the product temperatures are more significant, and the moisture content is lower (Mayor and Sereno 2004). Gomathi Padma Priya et al. (2024) observed similar trends in drying onions, noting that shrinkage values were at their minimum and maximum at temperatures of 60°C and 90°C, respectively, and during periods of 30 and 360 min. The shrinkage (%) is quantified as the change in product volume due to dehydration (Abbasi et al. 2009). The heat applied to food material during moisture removal is directly proportional to drying time and temperature, which decreases the sample's moisture content. The stresses in minced onions lead to contraction, intensifying the shrinkage. Shrinkage increases with rising temperature and time; however, in the final drying stages, the shrinkage value becomes stable due to structural stabilization from developing a hardened layer on the minced onions (El‐Mesery and Elabd 2021).

Equation (46) can be utilized to predict the (*S*_r_) of dried onions throughout the infrared drying process, considering the specific infrared power (*I*) and airflow (*V*) employed.

(46) $$S_{r}=\frac{0.32+0.031\;V-0.062\;V^{2}-0.88\;I}{1-0.17\;V-0.25\;V^{2}-1.31\;I-3.22\;I^{2}}\;R^{2}=0.999$$

The predictive Equation (47) can be utilized to estimate the (*S*_r_) of the sample throughout the infrared drying process, taking into account the (*I*) and (Th) employed.

(47) $$S_{r}=\frac{0.170-0.030\;\ln\mathrm{Th}+0.15\ln I+0.028\;{\left(\ln I\right)}^{2}}{1-0.033\ln\mathrm{Th}-0.061{\left(\ln\mathrm{Th}\right)}^{2}+1.030\ln I+0.24{\left(\ln I\right)}^{2}\;}\;R^{2}=0.999$$

### 3.9 Rehydration Ratio

**FIGURE 11:**
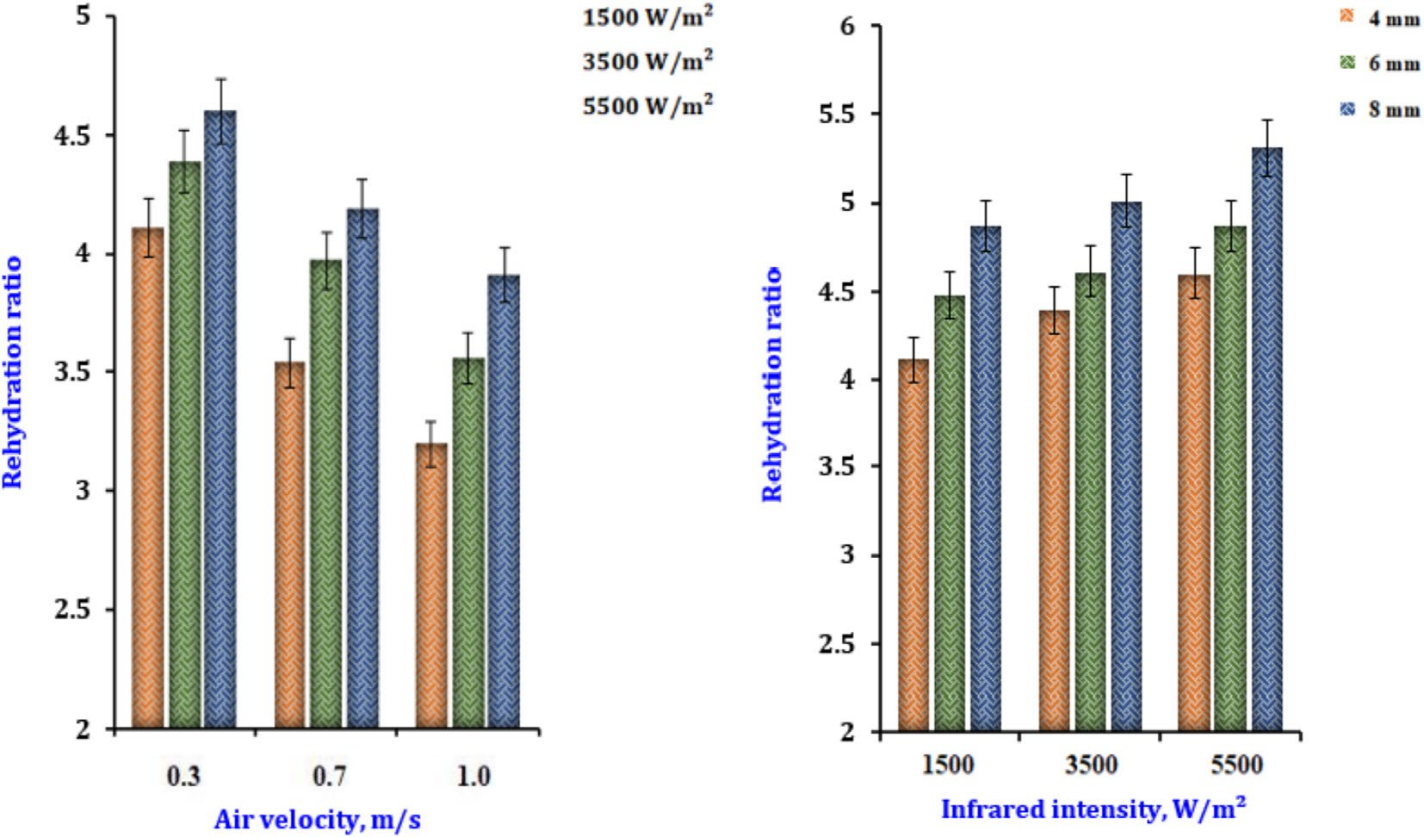
The effect of infrared radiation intensity and slice thickness on the rehydration ratio of dried onion.

Onion slices that have been dry in the past are shown in Figure 11. The rehydration ratio is shown after the slices have been dried using various drying air velocities and infrared radiation intensities. The dryer reaches its lowest value of 3.5 when operating at the lightest airflow and the greatest IR, and it reaches its highest value of 5.1 when operating at the shallowest airflow and the highest IR. With an increase in the IR, it is evident that the rehydration ratio drops as the airflow increases. Regarding changes in the rehydration ratio, increasing or decreasing the air velocity has a higher influence than changing the radiation intensity. It is possible to argue that the objects heated up more rapidly when exposed to greater infrared intensities, accelerating the generation of vapor inside the object and increasing its porosity (Kocabiyik and Tezer 2009).

The regression analysis revealed a correlation among the (*R*_r_), (*I*), and (*V*), as expressed by Equation (47).

(47) $$R_{r}=\frac{4.096-4.46\;V+1.46\;V^{2}+1.45\;I}{1-0.948\;V+0.29\;V^{2}-1.65\;I+1.006\;I^{2}}\;R^{2}=0.999$$

The thickness was determined to be between 4 and 8 mm, and the power was 0.34 W/cm^2^. The airflow was 0.3 m/s. In contrast to the onion with a thickness of 4 mm, the (*R*_r_) with thicknesses of 6–8 mm was found to be 53% and 35% more significant, respectively. It may be deduced from this that the (*R*_r_) was directly proportional to the thickness of the slice and that it grew with thickness (Elicin and Sacilik 2005).

Multivariate regression analysis (Equation 48) found a relationship between the power, thickness, and rehydration ratio.

(48) $$R_{r}=\frac{4.52-0.38\;\mathrm{Th}+4.40\;\ln I+1.21\;{\left(\ln I\right)}^{2}}{1-0.101\;\mathrm{Th}+0.0031\;{\mathrm{Th}}^{2}+0.10\;\ln I+0.30\;{\left(\ln I\right)}^{2}}\;R^{2}=0.996$$

## 4 Conclusions

This study investigated the influence of slice thickness, airflow, infrared power drying behavior, and onions' physico‐quality parameters. The drying period rises with rising airflow, independent of IR. The findings demonstrate that water activity generated by the IR drying process exhibits adequate stability for storage. The experiment's findings indicate that the model proposed by “Midilli and Kucuk” most effectively elucidates the drying process of onion slices in a thin layer. In the experimental infrared intensity range of 1500–5500 W/m^2^, the practical moisture diffusivity values varied across different experimental conditions, from 2.6 to 8.9 × 10^−10^ m^2^/s. With increasing slice thickness and infrared light intensity, moisture diffusivity decreased as airflow velocity rose. As airflow and slice thickness increased and infrared intensity decreased, the activation energy (*E*_a_) also improved. With a decrease in airflow and an increase in the rehydration ratio, there was a corresponding rise in thickness and infrared power. With an increase in airflow, the *S*_r_ exhibited an upward trend; conversely, an increase in slice thickness and infrared intensity resulted in a decline. The color difference increased with the onion slice's thickness and the intensity of the infrared light. The degree of browning is primarily due to color changes induced by Maillard reactions in onions. The brightness index (BI) rises with an increase in infrared power. Controlling infrared power and airflow enhances drying qualities and browning efficiency.

## Author Contributions

**Hany S. El‐Mesery:** conceptualization (equal), data curation (equal), formal analysis (equal), validation (equal), writing – original draft (equal). **Ahmed H. ElMesiry:** data curation (equal), formal analysis (equal), methodology (equal), validation (equal), visualization (equal), writing – original draft (equal), writing – review and editing (equal). **Zicheng Hu:** methodology (equal), project administration (equal), resources (equal), supervision (equal), validation (equal), visualization (equal), writing – review and editing (equal). **Xinai Zhang:** formal analysis (equal), investigation (equal), software (equal). **Evans K. Quaye:** data curation (equal), formal analysis (equal), investigation (equal), project administration (equal), supervision (equal), writing – original draft (equal), writing – review and editing (equal).

## Conflicts of Interest

The authors declare no conflicts of interest.

## Data Availability

The original contributions presented in the study are included in the article; further inquiries can be directed to the first author (Hany S. El‐Mesery, elmesiry@ujs.edu.cn) and the corresponding author.

## References

[fsn370437-bib-0001] Abbasi, S. , S. M. Mousavi , M. Mohebi , and S. Kiani . 2009. “Effect of Time and Temperature on Moisture Content, Shrinkage, and Rehydration of Dried Onion.”

[fsn370437-bib-0002] AOAC . 2005. Official Method of Analysis. 18th ed. AOAC Press. 10.32741/fihb.3.honey.

[fsn370437-bib-0003] Baptestini, F. M. , P. C. Corrêa , G. H. H. de Oliveira , L. F. J. Almeida , and G. A. Vargas‐Elías . 2016. “Constant and Decreasing Periods of Pineapple Slices Dried by Infrared.” Revista Brasileira de Ciências Agrárias 11: 53–59.

[fsn370437-bib-0004] Beigi, M. , H. B. Harchegani , M. Torki , et al. 2022. “Experimental and Numerical Analysis of Thermodynamic Performance of Microwave Dryer of Onion.” Journal of Food Process Engineering 45: e14116.

[fsn370437-bib-0005] Botelho, F. M. , P. C. Corrêa , A. Goneli , M. A. Martins , F. E. A. Magalhães , and S. C. Campos . 2011. “Periods of Constant and Falling‐Rate for Infrared Drying of Carrot Slices.” Revista Brasileira de Engenharia Agricola e Ambiental 15: 845–852.

[fsn370437-bib-0006] Castillo‐Téllez, M. , I. Pilatowsky‐Figueroa , E. C. López‐Vidaña , O. Sarracino‐Martínez , and G. Hernández‐Galvez . 2017. “Dehydration of the Red Chilli ( *Capsicum annuum* L., Costeño) Using an Indirect‐Type Forced Convection Solar Dryer.” Applied Thermal Engineering 114: 1137–1144. 10.1016/j.applthermaleng.2016.08.114.

[fsn370437-bib-0007] Chen, Q. , H. Song , J. Bi , et al. 2019. “Multi‐Objective Optimization and Quality Evaluation of Short‐ and Medium‐Wave Infrared Radiation Dried Carrot Slices.” International Journal of Food Engineering 15. 10.1515/ijfe-2018-0234.

[fsn370437-bib-0008] Darvishi, H. , A. R. Asl , A. Asghari , M. Azadbakht , G. Najafi , and J. Khodaei . 2014. “Study of the Drying Kinetics of Pepper.” Journal of the Saudi Society of Agricultural Sciences 13: 130–138. 10.1016/j.jssas.2013.03.002.

[fsn370437-bib-0009] Demiray, E. , and Y. Tulek . 2017. “The Effect of Pretreatments on Air Drying Characteristics of Persimmons.” Heat and Mass Transfer 53: 99–106. 10.1007/s00231-016-1797-2.

[fsn370437-bib-0010] Doymaz, I. 2012. “Drying of Pomegranate Seeds Using Infrared Radiation.” Food Science and Biotechnology 21: 1269–1275. 10.1007/s10068-012-0167-1.

[fsn370437-bib-0011] Elicin, A. K. , and K. Sacilik . 2005. “An Experimental Study for Solar Tunnel Drying of Apple.” Tarim Bilimleri Dergisi 11: 207–211.

[fsn370437-bib-0012] El‐Mesery, H. S. 2022. “Improving the Thermal Efficiency and Energy Consumption of Convective Dryer Using Various Energy Sources for Tomato Drying.” Alexandria Engineering Journal 61: 10245–10261.

[fsn370437-bib-0013] El‐Mesery, H. S. , K. Ashiagbor , Z. Hu , and M. Rostom . 2024. “Mathematical Modeling of Thin‐Layer Drying Kinetics and Moisture Diffusivity Study of Apple Slices Using Infrared Conveyor‐Belt Dryer.” Journal of Food Science 89: 1658–1671. 38317418 10.1111/1750-3841.16967

[fsn370437-bib-0014] El‐Mesery, H. S. , and M. A. Elabd . 2021. “Effect of Microwave, Infrared, and Convection Hot‐Air on Drying Kinetics and Quality Properties of Okra Pods.” International Journal of Food Engineering 17: 909–926. 10.1515/ijfe-2021-0125.

[fsn370437-bib-0015] El‐Mesery, H. S. , F. Sarpong , W. Xu , and M. A. Elabd . 2022. “Design of Low‐Energy Consumption Hybrid Dryer: A Case Study of Garlic (*Allium sativum*) Drying Process.” Case Studies in Thermal Engineering 33: 101929. 10.1016/j.csite.2022.101929.

[fsn370437-bib-0016] Gomathi Padma Priya, P. , S. Savitha , S. Chakraborty , and B. N. Thorat . 2024. “Effect of Dehydration and Pulsed Light Treatment on Decontamination of Minced Onions: Microbial Safety and Physicochemical Properties.” Journal of Food Science 89: 2025–2039. 38465674 10.1111/1750-3841.16990

[fsn370437-bib-0017] Hadibi, T. , D. Mennouche , A. Boubekri , et al. 2023. “Drying Characteristic, Sustainability, and 4E (Energy, Exergy, and Enviro‐Economic) Analysis of Dried Date Fruits Using Indirect Solar‐Electric Dryer: An Experimental Investigation.” Renewable Energy 218: 119291.

[fsn370437-bib-0018] Hu, Z. , Y. Li , H. S. El‐Mesery , D. Yin , H. Qin , and F. Ge . 2022. “Design of New Heat Pump Dryer System: A Case Study in Drying Characteristics of Kelp Knots.” Case Studies in Thermal Engineering 32: 101912. 10.1016/j.csite.2022.101912.

[fsn370437-bib-0019] Jiang, N. , C. Liu , D. Li , et al. 2017. “Evaluation of Freeze Drying Combined With Microwave Vacuum Drying for Functional Okra Snacks: Antioxidant Properties, Sensory Quality, and Energy Consumption.” LWT‐ Food Science and Technology 82: 216–226.

[fsn370437-bib-0020] Kocabiyik, H. , and D. Tezer . 2009. “Drying of Carrot Slices Using Infrared Radiation.” International Journal of Food Science and Technology 44: 953–959. 10.1111/j.1365-2621.2008.01767.x.

[fsn370437-bib-0021] Kumar, D. G. P. , H. U. Hebbar , D. Sukumar , and M. N. Ramesh . 2005. “Infrared and Hot‐Air Drying of Onions.” Journal of Food Processing and Preservation 29: 132–150. 10.1111/j.1745-4549.2005.00019.x.

[fsn370437-bib-0022] Lewis‐Atwell, T. , P. A. Townsend , and M. N. Grayson . 2022. “Machine Learning Activation Energies of Chemical Reactions.” Wiley Interdisciplinary Reviews: Computational Molecular Science 12: e1593.

[fsn370437-bib-0023] Li, L. , M. Zhang , and W. Wang . 2020. “Ultrasound‐Assisted Osmotic Dehydration Pretreatment Before Pulsed Fluidized Bed Microwave Freeze‐Drying (PFBMFD) of Chinese Yam.” Food Bioscience 35: 100548.

[fsn370437-bib-0024] Mayor, L. , and A. M. Sereno . 2004. “Modelling Shrinkage During Convective Drying of Food Materials: A Review.” Journal of Food Engineering 61: 373–386. 10.1016/S0260-8774(03)00144-4.

[fsn370437-bib-0025] Osae, R. , G. Essilfie , R. N. Alolga , E. Bonah , H. Ma , and C. Zhou . 2020. “Drying of Ginger Slices—Evaluation of Quality Attributes, Energy Consumption, and Kinetics Study.” Journal of Food Process Engineering 43: e13348.

[fsn370437-bib-0026] Ozyalcin, Z. O. , A. S. Kipcak , and N. Tugrul . 2023. “The Effect of Various Methods on the Drying Kinetics and Mathematical Modelling of Seabass (*Dicentrarchus labrax*).” Journal of Aquatic Food Product Technology 32: 384–395.

[fsn370437-bib-0027] Pekke, M. A. , Z. Pan , G. G. Atungulu , G. Smith , and J. F. Thompson . 2013. “Drying Characteristics and Quality of Bananas Under Infrared Radiation Heating.” International Journal of Agricultural and Biological Engineering 6: 58–70.

[fsn370437-bib-0028] Puente‐Díaz, L. , K. Ah‐Hen , A. Vega‐Gálvez , R. Lemus‐Mondaca , and K. Di Scala . 2013. “Combined Infrared‐Convective Drying of Murta (*Ugni molinae* Turcz) Berries: Kinetic Modeling and Quality Assessment.” Drying Technology 31: 329–338. 10.1080/07373937.2012.736113.

[fsn370437-bib-0029] Rajoriya, D. , S. R. Shewale , M. L. Bhavya , and H. U. Hebbar . 2020. “Far Infrared Assisted Refractance Window Drying of Apple Slices: Comparative Study on Flavour, Nutrient Retention and Drying Characteristics.” Innovative Food Science and Emerging Technologies 66: 102530. 10.1016/j.ifset.2020.102530.

[fsn370437-bib-0031] Royen, M. J. , A. W. Noori , and J. Haydary . 2020. “Experimental Study and Mathematical Modeling of Convective Thin‐Layer Drying of Apple Slices.” PRO 8: 1–17. 10.3390/pr8121562.

[fsn370437-bib-0032] Sacilik, K. , and A. K. Elicin . 2006. “The Thin Layer Drying Characteristics of Organic Apple Slices.” Journal of Food Engineering 73: 281–289. 10.1016/j.jfoodeng.2005.03.024.

[fsn370437-bib-0033] Sadin, R. , G.‐R. Chegini , and H. Sadin . 2014. “The Effect of Temperature and Slice Thickness on Drying Kinetics Tomato in the Infrared Dryer.” Heat and Mass Transfer 50: 501–507.

[fsn370437-bib-0034] Salehi, F. 2020a. “Recent Advances in the Modeling and Predicting Quality Parameters of Fruits and Vegetables During Postharvest Storage: A Review.” International Journal of Fruit Science 20: 506–520.

[fsn370437-bib-0035] Salehi, F. 2020b. “Recent Applications and Potential of Infrared Dryer Systems for Drying Various Agricultural Products: A Review.” International Journal of Fruit Science 20: 586–602. 10.1080/15538362.2019.1616243.

[fsn370437-bib-0036] Salehi, F. , K. Goharpour , and H. R. Kamran . 2023. “Effects of Ultrasound and Microwave Pretreatments of Carrot Slices Before Drying on the Color Indexes and Drying Rate.” Ultrasonics Sonochemistry 101: 106671. 37918296 10.1016/j.ultsonch.2023.106671 PMC10643527

[fsn370437-bib-0037] Salehi, F. , M. Inanloodoghouz , and M. Amiri . 2023. “Effect of Sonication and Edible Coating on Total Phenolic Content, Antioxidant Capacity, and Physical Characteristics of Infrared‐Dried Sweet Cherries.” Journal of Food Processing & Preservation 2023: 5014055.

[fsn370437-bib-0038] Salehi, F. , and M. Satorabi . 2021. “Influence of Infrared Drying on Drying Kinetics of Apple Slices Coated With Basil Seed and Xanthan Gums.” International Journal of Fruit Science 21: 519–527.

[fsn370437-bib-0040] Singh, S. , and S. Kumar . 2012. “New Approach for Thermal Testing of Solar Dryer: Development of Generalized Drying Characteristic Curve.” Solar Energy 86: 1981–1991.

[fsn370437-bib-0041] Sonmete, M. H. , H. O. Mengeş , C. Ertekin , and M. M. Özcan . 2017. “Mathematical Modeling of Thin Layer Drying of Carrot Slices by Forced Convection.” Journal of Food Measurement and Characterization 11: 629–638. 10.1007/s11694-016-9432-y.

[fsn370437-bib-0042] Tuly, S. S. , M. Mahiuddin , and A. Karim . 2023. “Mathematical Modeling of Nutritional, Color, Texture, and Microbial Activity Changes in Fruit and Vegetables During Drying: A Critical Review.” Critical Reviews in Food Science and Nutrition 63: 1877–1900. 34459302 10.1080/10408398.2021.1969533

[fsn370437-bib-0043] Velić, D. , M. Bilić , S. Tomas , M. Planinić , A. Bucić‐Kojić , and K. Aladić . 2007. “Study of the Drying Kinetics of “Granny Smith” Apple in Tray Drier.” Agriculturae Conspectus Scientificus 72: 323–328.

[fsn370437-bib-0044] Wu, B. , X. Guo , Y. Guo , H. Ma , and C. Zhou . 2021. “Enhancing Jackfruit Infrared Drying by Combining Ultrasound Treatments: Effect on Drying Characteristics, Quality Properties and Microstructure.” Food Chemistry 358: 129845. 10.1016/j.foodchem.2021.129845. 33933954

[fsn370437-bib-0045] Ye, L. , H. S. El‐Mesery , M. M. Ashfaq , Y. Shi , H. Zicheng , and W. G. Alshaer . 2021. “Analysis of Energy and Specific Energy Requirements in Various Drying Process of Mint Leaves.” Case Studies in Thermal Engineering 26: 101113. 10.1016/j.csite.2021.101113.

[fsn370437-bib-0046] Younas, S. , Y. Mao , C. Liu , W. Liu , T. Jin , and L. Zheng . 2021. “Efficacy Study on the Non‐Destructive Determination of Water Fractions in Infrared‐Dried Lentinus Edodes Using Multispectral Imaging.” Journal of Food Engineering 289: 110226. 10.1016/j.jfoodeng.2020.110226.

[fsn370437-bib-0047] Zeng, S. , B. Wang , D. Zhao , and W. Lv . 2023. “Microwave Infrared Vibrating Bed Drying of Ginger: Drying Qualities, Microstructure and Browning Mechanism.” Food Chemistry 424: 136340. 37220685 10.1016/j.foodchem.2023.136340

[fsn370437-bib-0048] Zeng, Y. , Y. Liu , J. Zhang , H. Xi , and X. Duan . 2019. “Effects of Far‐Infrared Radiation Temperature on Drying Characteristics, Water Status, Microstructure and Quality of Kiwifruit Slices.” Journal of Food Measurement and Characterization 13: 3086–3096. 10.1007/s11694-019-00231-3.

[fsn370437-bib-0049] Zhang, W. , K. Wang , and C. Chen . 2022. “Artificial Neural Network Assisted Multiobjective Optimization of Postharvest Blanching and Drying of Blueberries.” Food 11: 1–18. 10.3390/foods11213347. PMC9659016 36359960

